# Sleep and Well-Being during the COVID-19 Remote and In-Person Periods: Experiences of College Faculty and Staff with and without Disabilities

**DOI:** 10.3390/bs13100844

**Published:** 2023-10-16

**Authors:** Catherine S. Fichten, Samantha Wing, Georgiana Costin, Mary Jorgensen, Alice Havel, Susie Wileman, Sally Bailes, Laura Creti, Eva Libman

**Affiliations:** 1 Department of Psychology, Dawson College, 3040 Sherbrooke St. W., Montreal, QC H3Z 1A4, Canada; 2 Department of Psychiatry, McGill University, 1033 Pine Avenue West, Montreal, QC H3A 1A1, Canada; sally.bailes@mcgill.ca (S.B.); lcreti@gmail.com (L.C.); eva.libman@mcgill.ca (E.L.); 3 Behavioural Psychotherapy and Research Unit, Jewish General Hospital, 4333 Cote Ste Catherine Road, Montreal, QC H3T 1E4, Canada; 4 Adaptech Research Network, 4001 de Maisonneuve W., Montreal, QC H3Z 3G4, Canada; samanthamayawing@gmail.com (S.W.); georgiana.costin@mail.mcgill.ca (G.C.); mjorgensen07@ubishops.ca (M.J.); ahavel@dawsoncollege.qc.ca (A.H.); swileman@dawsoncollege.qc.ca (S.W.); 5 McGill Cognitive Science Program, McGill University, 845 Rue Sherbrooke O, Montréal, QC H3A 0G4, Canada; 6 Department of Psychology, McGill University, 845 Rue Sherbrooke O, Montréal, QC H3A 0G4, Canada; 7 Scholars in Residence Program, Dawson College, 3040 Sherbrooke St. W., Montreal, QC H3Z 1A4, Canada

**Keywords:** COVID-19, sleep, well-being, remote and in-person work, faculty and staff, disabilities

## Abstract

We explored the impacts of the remote and return-to-in-person work periods on sleep and well-being as reported by faculty (*n* = 22) and non-teaching staff (*n* = 21) with and without disabilities. Participants were recruited through college platforms and personal contacts. Our results show that contrary to expectations, the COVID-19 remote teaching/working period resulted in better sleep, as well as greater well-being, than the return-to-in-person work period. With respect to sleep, faculty members had slightly more negative outcomes than staff, most evident in heightened anxiety and work aspects. Faculty with disabilities had somewhat worse sleep and well-being during the remote period than faculty without disabilities. During the return to in-person work, both faculty and non-teaching staff reported more negative than positive sleep and well-being outcomes. In particular, during the in-person period, faculty members experienced slightly more negative sleep outcomes related to anxiety and work, while staff members experienced slightly more negative sleep outcomes related to the need to commute and lifestyle. Our findings show that there were benefits and disadvantages to both remote and in-person work periods, suggesting a hybrid work schedule should be considered in more detail, particularly as an optional reasonable accommodation for faculty and staff with disabilities. Our study highlights that training to keep faculty abreast of the latest technological innovations, ways to promote work–life balance, and steps to remedy classroom size and building ventilation to prevent the spread of disease all need urgent attention.

## 1. Introduction

The COVID-19 pandemic officially began in March 2020 in Canada and sent much of the country home. With the exception of essential services, many jobs shifted to remote platforms, and virtually all junior/community colleges in Quebec, Canada’s second largest province (approximately 22% of the population), shifted to remote teaching and working to limit the transmission of the virus [1]. Instructors, most of whom had no experience with remote teaching, had to learn how to teach online within two weeks of the pandemic starting. Non-teaching staff also had to pivot to remote work. The restrictive public health measures and social isolation during the COVID-19 remote period affected the well-being of many individuals [2,3]. Aspects such as work–life balance, housing conditions, and health status were all affected [4]. Additionally, the stress and anxiety about contracting the virus, or about a loved one getting sick, affected the sleep and well-being of innumerable Canadians [5,6].

Teaching and work officially returned to the in-person mode in Quebec’s junior/community colleges in Fall 2021, after a year and a half online. Faculty and non-teaching staff had very different professional lives. For example, faculty returned to 12–18 h of teaching per week between 8 a.m. and 6:00 p.m. Faculty could specify their office hours. Non-academic staff typically worked a 35-h work-week between 9 a.m. and 5 p.m. with an hour off for lunch. With the COVID-19 virus still circulating at that time, wearing masks and obeying social distancing guidelines were mandatory when returning to in-person teaching and working in colleges [7,8]. This transition was difficult for many people after working and teaching remotely for so long [9].

### 1.1. Sleep Experiences during the Remote Period

A systematic review and meta-analysis showed that around the world 4 out of 10 individuals experienced sleep disturbances during the COVID-19 pandemic [10], with poor sleep quality and insomnia being the primary complaints [5,10]. While sleep duration increased during the COVID-19 remote period due to a lack of commuting and increased flexibility in schedules [11], sleep quality suffered [12,13] as a result of increased anxiety and stress, as well as an overall disrupted routine [13,14]. Furthermore, there was an increased reliance on technology to teach, work, and stay connected during the COVID-19 remote period. Therefore, sleep quality may have also been affected by the increase in technology use as screen exposure in the hours before falling asleep is associated with reduced sleep quality [14,15,16]. 

While most studies highlight sleep disturbances such as insomnia and poor sleep quality during the COVID-19 remote period, some reports show that favorable sleep outcomes were also experienced. This was attributed to the ability to shift sleep schedule as desired, because of increased flexibility in bedtime and arising times [11]. 

### 1.2. Disability

There are few studies regarding the impact of the remote and in-person periods on individuals with disabilities such as chronic health conditions, attention deficit hyperactivity disorder, or sensory disabilities such as low vision. Generally, studies that we were able to find focused on factors such as pain syndromes [17,18]. For example, Çiftçi and Demirhan (2023) found that the health-related quality of life among those with neck pain was worse among people working remotely than among those working in person [19]. 

Beckel et al. (2022), in a conceptual article, noted that remote work could—and should—be considered a disability accommodation since some individuals with disabilities can have fluctuating energy levels while others have difficulty attending classes during icy conditions and snow storms [20]. This view is consistent with our interest and hypotheses concerning sleep and well-being during the remote and return-to-in-person periods among post-secondary employees who have a disability. 

### 1.3. Remote Period 

The COVID-19 pandemic demanded a transition to online learning for many professors as well as non-teaching post-secondary staff. Working and learning remotely were major shifts in the daily routine for many. For some instructors, the transition to remote teaching was accompanied by stress and anxiety about adapting to a new teaching method, particularly new technology [11,21]. The online platform can also be challenging for instructors in terms of student engagement and attendance. Speaking to “the void” arose as a psychological challenge for many instructors during the COVID-19 remote period [21]. The transition to remote work likely varied in difficulty depending on factors such as family situations and living arrangements, level of comfort with technology, and general lifestyle. In addition, remote work can also lead to reduced social interactions during the day. With the COVID-19 virus limiting in-person social interactions during leisure time, well-being was negatively affected for many individuals [22]. Furthermore, decreased mobility due to the COVID-19 restrictions, as well as the shift to working on an online platform, resulted in decreased physical activity for many people; this, too, can have adverse effects on sleep and well-being [23].

Al Miskry et al. (2021) conducted a study comparing the effects of the COVID-19 lockdown on university faculty, staff, and students in the UAE [24]. They found that faculty and students experienced greater levels of distress than non-teaching staff. Additionally, they found that women experienced higher levels of distress than men during this time. Supporting these findings, a study conducted in Ireland found that teachers, including primary, secondary, and post-secondary teachers, experienced higher levels of burnout and stress during the pandemic as compared to the pre-pandemic period [25].

### 1.4. Return to In-Person Work

As the COVID-19 virus gradually became less of a threat, with the number of cases decreasing and access to vaccines increasing, the world had to re-establish its working and learning routines. While some post-secondary employees fully returned to the in-person routine, others stayed entirely remote or adopted a hybrid schedule. A longitudinal study of sleep disturbances and mental health among a population of 1062 Italians found a decrease in sleep disturbances, insomnia, depression, and anxiety symptoms upon return to in-person work [26]. However, these improvements were relatively small, and both Salfi et al. and Massar et al. (2021) reported a decrease in sleep duration with the re-establishment of in-person work and school routines [16,23]. This is most likely a consequence of having to wake up earlier in order to commute. The return to in-person work also came with an increased risk of COVID-19 transmission, which was likely anxiety-inducing for the general population [27]. As colleges accommodate thousands of people, it was stressful for faculty, staff, and students to return to face-to-face activities in Fall 2021 with the COVID-19 virus still circulating and with the need to follow restrictions (e.g., social distancing, wearing a mask, etc.) [8].

Massar et al. (2021) found that, in a sample of 200 staff and students from the University of Singapore, the return to in-person activities had some positive results, with an increase in physical activity due to the routine of going to work and the loosening of restrictions, increasing activity in general [23]. Social interaction, another lifestyle factor impacted during the remote period of the COVID-19 pandemic, almost fully returned to normal with the return to in-person activities [28]. Although this increase in social interactions came with anxiety about COVID-19 transmission, positive well-being outcomes remained. 

Ozamiz-Etxebarria et al. (2021) examined the experiences of 1633 teachers, including post-secondary instructors, during the return to in-person teaching activities and found that a high percentage were suffering from stress and anxiety during this time [27]. This study and others show that reduced well-being outcomes are not only a result of the COVID-19 remote period but also a consequence of the return to in-person activities [9].

## 2. Present Study

To the best of our knowledge, this is the first study to compare sleep and well-being during the remote and in-person periods of academic staff and faculty with and without disabilities. While there are studies comparing remote to in-person work experiences, many of these compared pre-COVID experiences with remote experiences [29,30,31]. Therefore, the aim of this primarily descriptive cross-sectional study is to explore sleep and subjective well-being [32] experiences of faculty and non-teaching staff with and without disabilities at a large metropolitan junior/community college in Quebec during the recent COVID-19 pandemic remote period as well as during the return-to-in-person periods. We tested the following hypotheses:

**H1.**  *We expected to see negative impacts (i.e., more negative well-being and sleep codes) of the COVID-19 remote period on the sleep and well-being of both staff and faculty, especially those without disabilities [11,21].*

**H2.**  *We hypothesized that faculty would experience more negative sleep and well-being outcomes than non-teaching staff during the remote period due to the challenges related to teaching with unfamiliar technology [33]*.

**H3.**  *We predicted that notwithstanding Hypothesis 1, staff with disabilities would have more favorable sleep and well-being experiences during the remote period than during the in-person period since remote work is often seen as a disability accommodation [20].*

**H4.**  *We expected to see positive impacts of the return to in-person routine on work- and lifestyle-related well-being, in both faculty and non-teaching staff (since work is sometimes more effective in person, and increased social interaction often has a positive effect on well-being).*

**H5.**  *However, we also expected some negative impacts of return to in-person work on sleep and well-being because of the return to commuting for many, as well as an increased risk of COVID-19 transmission, especially for faculty.*

## 3. Method

### 3.1. Sample

Participants consisted of 43 individuals, comprising 22 faculty (10 with a disability) and 21 non-teaching staff (10 with a disability) working at a large metropolitan junior/community college in Quebec. All had been working at their current job for at least three years, ensuring that they had experienced both the COVID-19 remote teaching/working period (from mid-March 2020 onwards) as well as the return to in-person teaching/working (Fall 2021). Notably, 81% of the study participants were female, and 19% were male. To the best of our knowledge, none had experienced a COVID-19 infection. Table 1 shows the age distribution of the study population, indicating that most participants were aged 45 and over. 

Notably, 50% of our sample comprised faculty and staff with disabilities. Their characteristics (see Table 2) show that the most common disability among our participants was a chronic health-related disability, followed by a sensory disability and attention deficit hyperactivity disorder. One third of the participants had two or more disabilities.

### 3.2. Measures

Demographic Information. We collected information about gender, age, and the presence or absence of a disability, as well as the type of disability. As recommended by AHEAD (2012) and Banerjee et al. (2020), we reported the disabilities disclosed by the participants [34,35] 

Sleep and Well-Being Measures. Three open-ended questions asked participants about the positive and negative factors that affected their sleep and well-being during the remote and in-person periods.

1. What were the positive and negative factors that affected your sleep and well-being during the remote teaching/working period?
2. What were the positive and negative factors that affected your sleep and well-being during the return to in-person teaching/working?
3. Is there anything else you would like to tell us about your sleep?

### 3.3. Procedure

During the remote period (March 2020 onwards), all education programs in Quebec’s junior/community colleges were held exclusively online, mainly via Zoom. Most non-teaching staff members also worked online. Starting in the Fall of 2021, staff and faculty returned to in-person work. 

The research protocol was approved by the Dawson College Research Ethics Board (Certificate #FICHC21224325). Participants were recruited during October and November of 2022 through college platforms and personal contacts. We emailed interested individuals an information and consent form as well as the demographic information questions. Those who completed these questions were invited to take part in one of four one-hour-long Zoom focus groups (two for faculty and two for non-teaching staff). If a participant could not attend a focus group, an interview was scheduled instead. This usually lasted approximately half an hour. Interviews were held with 20 participants (12 faculty and 8 staff). 

Zoom focus groups and interviews were not recorded. Two research team members took notes during the focus groups, and these notes were then combined. One team member took notes during interviews. As a token of our appreciation, each participant received a CA$ 30 Amazon gift card. 

A coding manual was developed to categorize participant responses [36], and group thematic coding was conducted [37] by three team members. All responses were coded into a positive or negative sleep or well-being category. In case of discrepancies, coders discussed their responses and agreed upon a consensus code. Once we coded the responses, we analyzed the frequencies of each category for both the remote and in-person periods (Table 3).

## 4. Results

**Figure 1:**
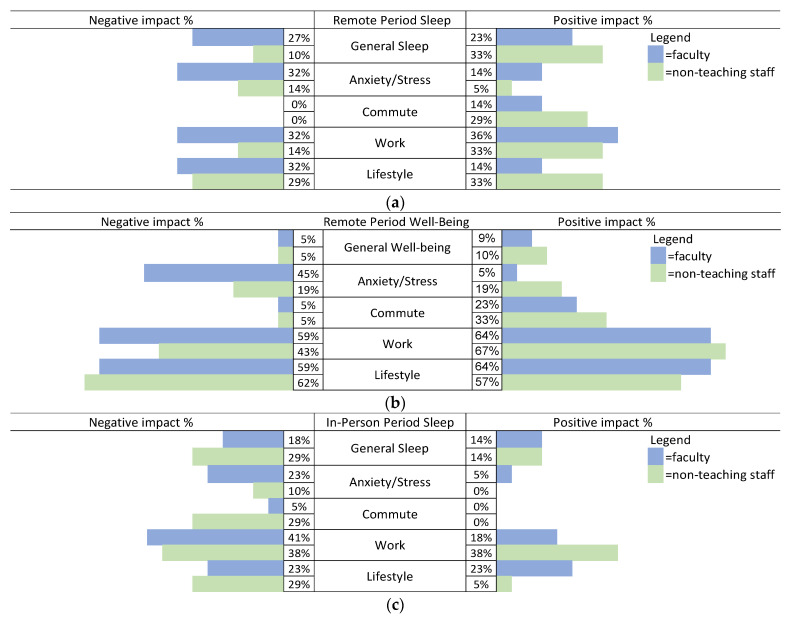

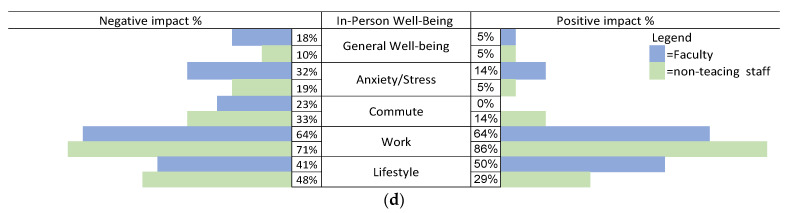
(**a**–**d**) Percentages calculated are based on 22 faculty and 21 staff.

To enable statistical analyses that reflect both positive and negative experiences, we converted the four frequency scores in Figure 1 to states-of-mind (SOM) ratios [38]. This involved dividing the number of positive codes by the sum of positive and negative codes, with a correction of 1 in case either the negative or positive frequency was 0. The larger the number, the more favorable the experience. As a check on SOM scores, we also carried out a series of chi-square tests on the frequencies related to Figure 1. 

### 4.1. Sleep and Well-Being Comparisons between Disability Status, Faculty vs. Staff, and Remote vs. In-Person Periods

**Sleep.** A 2 (in-person/remote) × 2 (positive/negative) chi-square test of sleep category frequencies showed that participants (*n* = 43) had relatively more negative and fewer positive sleep-related comments during the in-person period than during the remote period (X^2^(1,168) = 8.53, *p* = 0.004). 

Table 4 and the results of a three-way mixed-design analysis of variance (ANOVA) (2 (disability/no disability) × 2 (faculty/staff) × 2 (remote/in-person)) on SOM sleep scores show a significant main effect of remote/in-person sleep (*F*(1,33) = 5.61, *p* = 0.024, η = 0.145). This indicates that the participants had higher SOM scores (i.e., better overall sleep) during the remote period (*M* = 0.504, *SD* = 0.156) than during the in-person period (*M* = 0.432, *SD* = 0.118). 

The three-way ANOVA interaction on SOM scores approached significance (*F*(1,33) = 3.66, *p* = 0.068, η = 0.097). Post hoc tests suggest that, during the remote period, the sleep of faculty was worse than that of staff. Figure 1a and the chi-square test results also show that, during the remote period, the sleep of faculty was significantly worse than that of non-teaching staff (*X*^2^(1,91) = 4.33, *p* = 0.038). Table 4 suggests that this is mainly because faculty with disabilities had somewhat worse scores during the remote period than the other three groups.

Post hoc tests on the SOM ratio also suggest that the sleep of staff without disabilities was worst during the in-person period and best during the remote period (*p* < 0.10). Indeed, the chi-square test results show that, during the in-person period, staff without disabilities had relatively fewer positive codes than those with disabilities (*X*^2^(1,40) = 3.25, *p* = 0.071) and relatively more negative codes than those with disabilities (*X*^2^(1,42) = 3.94, *p* = 0.047).

**Well-Being**. A 2 (in-person/remote) × 2 (positive/negative) chi-square test of well-being frequencies shows that participants (*n* = 43) had somewhat more negative and fewer positive well-being comments during the in-person period than during the remote period (*X*^2^(1,276) = 2.89, *p* = 0.089) (i.e., better overall well-being during the remote period). 

Table 5 and the results of a three-way mixed-design ANOVA (2 (disability/no disability) × 2 (faculty/staff) × 2 (remote /in-person)) on SOM well-being scores shows only a significant remote/in-person main effect (*F*(1,35) = 6.50, *p* = 0.015, *η* = 0.157). This indicates that the participants had higher SOM scores (i.e., better overall well-being) during the remote period (*M* = 0.515, *SD* = 0.165) than during the in-person period (*M* = 0.431, *SD* = 0.152). 

The three-way interaction on SOM scores again approached significance (*F*(1,35) = 3.27, *p* = 0.077, η = 0.087). None of the post hoc tests were significant. The remote scores in Table 5 suggest that the well-being of faculty with disabilities appears to be worse than that of the other three groups and that the well-being of staff with disabilities appears to be worse than that of the other three groups during the in-person period.

### 4.2. Remote Period: Positive and Negative Impacts on Sleep and Well-Being in the Five Categories Reported by Faculty and Staff

***Sleep during the remote period.*** Consistent with the SOM ratios and chi-square test results, Figure 1a shows that, overall, during the remote period, faculty reported more negative impacts on their sleep in all categories than non-teaching staff did. 

### 4.3. Remote Period: Illustrative Examples of Positive and Negative Impacts on Sleep Responses per Category for Faculty and Staff 

#### Negative Aspects of Sleep during the Remote Period

Anxiety/stress category: I couldn’t sleep—I experienced plenty of anxiety and lost sleep worrying about the long-term impacts of the pandemic in Canada and abroad; concerns about COVID transmission affected my sleep.

Work category: Feeling nervous about technology in the transition to online kept me up at night; I worried about technology—trying to get software installed—technicalities would wake me up in the middle of the night. 

Lifestyle category: Sleep-wise, COVID wasn’t the factor—worrying about mom’s health kept me up at night; I couldn’t exercise the way I usually do; this negatively affected my sleep and mental health.

*Positive aspects of sleep during the remote period*. There were similar positive sleep outcomes for faculty and non-teaching staff during the remote period. 

Anxiety/stress category: I had less anxiety during COVID—slept well; I could sleep well knowing I was home.

Work category: When working from home, I got extra sleep time; I experienced the luxury of waking up, rolling over, and teaching directly after waking up.

Lifestyle category: I could get up early enough that I could spend an hour with a cup of coffee and go for a walk outside; I could take a nap during the day—the COVID period provided for this flexibility.

### 4.4. Illustrative Examples of Positive and Negative Impacts on Well-Being Responses per Category for Faculty and Staff

As Figure 1b,d show, overall, there were a larger number of comments on well-being than on sleep during both the remote and in-person periods. 

### 4.5. Remote Period: Illustrative Examples of Positive and Negative Impacts on Well-Being Responses Per Category

*Negative aspects of well-being during the remote period*. As Figure 1b suggests, there were somewhat fewer negative than positive comments. The one category with more negative responses, especially by faculty, is the anxiety/stress category. 

Anxiety/stress category: Biggest factor was stress because we did not know what was happening; COVID was a big factor in anxiety—all of the precautions, etc.

Other negative comments are indicated below.

Work category: I felt nervous about technology in the transition to online; teaching to a bunch of black screens was like speaking into a void.

Lifestyle category: I used to sit and worry for hours, and doom scroll on computers during the pandemic; I’m a social person, so being away from people felt isolating and difficult.

*Positive well-being experiences during the remote period*. As the SOM ratios and the results in Figure 1b show, there were many positive comments as well. Other than the commute category, this was mainly true of the work and lifestyle categories. 

Lifestyle category: Going out for walks was positive—this became a habit; I have found more productive uses of my time.

Work category: I appreciated the flexibility of being in control of my schedule; when I was in remote work, the house was cleaner.

### 4.6. In-Person Period: Positive and Negative Impacts in the Five Categories on Sleep and Well-Being Reported by Faculty and Staff

***Sleep during the in-person period***. Consistent with the SOM ratio results, Figure 1c also shows that the sleep experiences of our study population during the return to in-person teaching/working were again more negative than positive. 

*Negative aspects of sleep during the in-person period*. As Figure 1c shows, more participants commented on the negative aspects of sleep than the positive aspects for every category. Again most comments about sleep during the return to in-person routine were related to work. 

### 4.7. In-Person Period: Illustrative Examples of Positive and Negative Impacts on Sleep Responses per Category

#### Negative Aspects of Sleep during the In-Person Period

Work category: Being back in person, my sleep was more disrupted overall; teaching in person comes with anxiety about waking up being late.

Of course, there were negative comments about having to travel to work, mainly by non-teaching staff.

Commute category: Always traveling and getting up early can affect you—I had less sleep because of that; elements of stress and problematic sleep because of commuting: one never knows if the metro will shut down, etc. 

Participants also indicated the negative impact of lifestyle and anxiety on sleep.

Lifestyle category: My priority on the weekend is sleep! But that takes away from other personal and household things—I have not found the balance; I would often find myself staying awake late into the night, and as such I frequently felt sleep-deprived.

Anxiety/stress category: The increased stress and anxiety from being back in person makes me lose sleep; I would wake up with the feeling I had in a dream; this was anxiety and fear.

*Positive impacts on sleep during the return to in-person work*. The return to in-person work also had positive impacts on sleep, which were mainly related to work and lifestyle:

Work category: Sleep is easier when I am working in person—getting out of the house; I am sleeping better now than during remote work.

Lifestyle category: I have no trouble falling asleep because I am exhausted from the day; sleeping less allows for peaceful activities like reading or tidying in the morning before my children wake up.

***Well-being during the in-person period.*** As was the case with sleep experiences, and consistent with the SOM ANOVA results, Figure 1d shows that the participants reported more negative than positive well-being outcomes during the return to in-person work. 

*Negative well-being experiences during the in-person period*. Again, the participants, especially faculty, had more negative than positive responses related to anxiety/stress. They also pointed to many negative, as well as positive, work- and lifestyle-related impacts on their well-being.

### 4.8. Remote Period: Illustrative Examples of Positive and Negative Impacts on Well-Being Responses per Category

Anxiety/stress category: More anxiety coming in person—I had a lot of unexpected meetings with policies changing and people getting COVID, etc.; concerns about COVID transmission.

Work: Concerns about breathing, never mind teaching, with the mask; Oh, the social anxiety going back to in-person teaching! 

Lifestyle: Socially tiring—I got used to being more of an introvert in COVID: I have to push back personal priorities to the weekend now that we’re in person.

*Positive well-being experiences during the in-person period*. Overall, 32 of the 43 participants commented on positive well-being outcomes related to work during the return to in-person routine, making it the most talked about category of the whole study. 

Work category: I was able to see the students’ direct feedback—I was jumping up and down, it was fabulous; once back at work, job was much easier to do on-site than remotely.

Lifestyle: Being able to socialize with colleagues and actually get to know them was very much appreciated; ability to do other things outside of school with COVID restrictions lifting is very important.

## 5. Discussion 

It is evident that there were both positive and negative impacts on sleep and well-being during both the COVID-19 remote teaching/working period, as well as during the return-to-in-person period. Both sleep and well-being were better during the remote than the in-person period. This does not validate part of Hypothesis 1, as we expected overall worse sleep and well-being during the remote period. 

Notably, 50% of our sample comprised faculty and staff with disabilities. In general, our results suggest that faculty with disabilities had somewhat worse experiences than their non-disabled counterparts when it came to sleep and well-being both during the remote and in-person periods. Our findings related to staff are less clear since their sleep and well-being results were not consistent. 

### 5.1. The Remote Period

As noted earlier, there were several differences between the tasks of faculty and staff and those seem to be reflected in their sleep and well-being. Staff generally had a “9 to 5” type of schedule, whereas faculty, who generally taught 12 to 15 h spread across the timetable and thus always had more flexibility. Also, faculty, most of whom had no previous experience with online or remote teaching, had to pivot to remote teaching during a two-week period. Although staff also needed to adapt to the remote environment, the nature of their tasks may have made this transition less onerous. 

Although the participants’ sleep was significantly better during the remote period than during the in-person period, this is not to say that their sleep was good; only that it was better than during the in-person period, where it was much worse. This was also true of well-being, where, in spite of a plethora of negative well-being findings reported in the literature for both faculty [11,21] and office workers [22], the well-being of our participants was also significantly more favorable during the remote period than during the in-person period (cf. Peacock). 

Consistent with Hypothesis 2, overall, during the remote period, the sleep of faculty was worse than that of staff. Also, consistent with the results of Al Miskry et al. (2021), in many instances, the well-being of faculty was also worse than that of staff [24]. Surprisingly, Hypothesis 3 was not validated as the sleep and well-being of faculty with disabilities were especially poor during the remote period. 

Others found worsened sleep quality, increased mood disturbance, and lower quality of life among office workers during the remote period in comparison to the “pre-COVID” experience [22]. Our comparison of remote and “post-COVID” (i.e., in-person) work showed that staff appeared to fare slightly better than faculty during the remote period, specifically in the form of fewer negative impacts on sleep. 

It is possible that the challenging nature of remote teaching, the sudden transition to an online platform such as Zoom, and having to learn how to use the technology account for some of the anxiety and stress that likely had a negative effect on the sleep and well-being of faculty members. Issues surrounding technology use were especially problematic for faculty who did not have any experience with online teaching. Faculty also complained about the demoralizing feeling of teaching to “a bunch of black screens”. Another important category of negative comments by faculty was related to the need to be available to students at all times, not allowing for work–life separation. For both faculty and staff, the remote period had serious impacts on lifestyle, often linked to social isolation. 

Although much of the literature discusses negative sleep and well-being-related impacts during the remote period, as noted by our participants, there were certainly some positives [11]. This includes having more time to sleep, feeling safer because of reduced exposure to the virus, and having a more flexible schedule. Positive impacts on lifestyle and work associated with flexibility included having control over one’s schedule; having more free time because there was no need to travel to work; and being able to engage in personal activities, such as exercising, walking, and gardening. For lifestyle, there appeared to be a very similar number of negative and positive impacts for both faculty and staff. On the negative side was social isolation, while on the positive side was more leisure time. 

In contrast to faculty, who experienced mainly negative impacts on their sleep during the remote period, non-teaching staff had somewhat more favorable experiences. In particular, they commented on the positive impact of not having to travel to work and having more favorable lifestyle experiences. Indeed, for non-teaching staff, particularly those without disabilities, the remote period had the most favorable impact on their sleep.

### 5.2. The Return-to-In-Person Period

The return to in-person teaching and work had a more significant negative effect on sleep and well-being outcomes for both faculty and staff than the remote period. The well-being of staff with disabilities appeared to be particularly poor during the in-person period. The cause of this latter finding is unclear, although several participants mentioned that the college refused their request for remote work some of the time.

*Sleep*. In fact, there were almost no positive impacts of the return to in-person teaching and work on sleep. As for well-being, consistent with Hypothesis 4, the main positives of the return to in-person work were in the realm of work and lifestyle. Faculty were pleased to see their students and enjoyed getting feedback from students in person. However, relatively more staff members reported positive impacts of work on their well-being than faculty. One of the positive work experiences mentioned was that performing one’s job in person was easier than remotely. Also, the ability to socialize with colleagues was especially important. A relatively large number of non-teaching staff commented on the positive sleep outcomes related to work, highlighting the mixed experiences during this period. 

As suggested in Hypothesis 5, there were also numerous negative well-being outcomes related to work and lifestyle. For example, faculty frequently reported performance anxiety upon returning to in-person teaching. In addition, the return to congested classrooms increased concern among faculty about catching COVID-19, regardless of wearing masks [7,8], as it was impossible to maintain social distancing. Non-teaching staff also had negative experiences. They were especially concerned about the negative impact of the commute on crowded buses and subways during rush hour. The commute time and the anxiety related to the possibility of COVID-19 transmission led to less and worse sleep and poorer well-being. 

### 5.3. Limitations 

We used a convenience sample, and our sample size was relatively small and did not constitute a random sample. Also, as in many other studies, our sample mainly consisted of female participants [24]. The order of sleep quality questions was constant, and responses concerning the remote period were retrospective. Both the remote and return-to-in-person periods were long, and there were likely differences throughout each period [26]. These can all affect the generalizability of our results.

## 6. Summary

Our results show that contrary to expectations, the COVID-19 remote teaching/working period resulted in better sleep, as well as greater well-being, than the return-to-in-person period. With respect to sleep, during the remote period, faculty members had slightly more negative outcomes than non-teaching staff; this was most evident in sleep disturbance due to heightened anxiety and difficulties with work. Faculty with disabilities had somewhat worse sleep and well-being during the remote period than faculty without disabilities.

During the return to in-person work, both faculty and non-teaching staff reported more negative than positive sleep and well-being outcomes. In particular, during the in-person period, faculty members were found to have slightly more negative sleep outcomes related to anxiety and work, while staff members revealed slightly more negative sleep outcomes related to the need to commute and lifestyle. 

Our findings show that there were benefits and disadvantages to both remote and in-person work periods, suggesting that a hybrid work schedule should be considered in more detail, particularly as an optional reasonable accommodation for faculty and staff with disabilities. Our study highlights that training to keep faculty abreast of the latest technological innovations, ways to promote work–life balance, and steps to remedy classroom size and building ventilation to prevent the spread of disease all need urgent attention.

## 7. Implications

Our findings are consistent with those of Abston and Soter (2023), who explored the residual negative effects of the remote period on the return to in-person teaching [9]. In a paper provocatively titled “Managing Expectations in a Pandemic and ‘Getting Back to Normal’”, they concluded that the “new normal” is not the old normal as faculty continue to experience negative stress and well-being outcomes. What lessons can be learned from our findings that can be used to enhance the sleep and well-being of both faculty and non-teaching staff in this “new normal” period? The stakeholders of which sectors need to become involved for change to occur?

Our results indicate that both sleep and well-being were better during the remote period than during the in-person period. This was true for both staff and faculty, even though staff generally have a “9 to 5” type of schedule, whereas faculty have always had more flexibility. Furthermore, according to our study, non-teaching staff can experience frustration when they must sit in their offices and field questions from students and teachers who are working remotely [39]. 

Faculty, most of whom had no previous experience with remote teaching, had to familiarize themselves with platforms such as Zoom and pedagogy suitable for online teaching. This steep learning curve resulted in anxiety and stress, which had a negative effect on sleep and well-being. Although great efforts were directed toward providing support for faculty during the crisis period, training to keep faculty abreast of the latest technological innovations must be continuous. Technology advances rapidly, and one cannot predict when the next crisis will occur.

Both faculty and staff reported that the remote period provided more time to engage in personal tasks, exercise, and be involved in other leisure activities. On the other hand, they also indicated that it did not allow for sufficient work–life separation. Perhaps human resources could play a role in developing policies to address these aspects of work–life balance: promoting more flexible work schedules; facilitating health-promoting activities like exercise within the workplace; or setting limits for work-related communication outside of work hours. 

Faculty anxiety upon return to in-person work heightened as it meant a return to congested classrooms and increased concern about COVID-19 infection. Overcrowded classrooms and poor air quality are not new, but they took on much greater significance during the remote COVID-19 period. There is no quick remedy for problematic classroom size and building ventilation; however, our findings highlight the need for a plan to be developed *now*, and steps to ameliorate this situation should begin as soon as possible.

As for policy implications, our findings suggest that hybrid arrangements, especially for non-teaching staff but also for faculty, may be an excellent option since a hybrid model would incorporate the best aspects of both worlds. The well-being of staff with disabilities was especially poor during the return to in-person work. Notably, several participants mentioned that the college refused requests to work remotely some of the time, and thus a hybrid work schedule might be a reasonable solution for them as well. It is important to consider the reasons behind the finding that faculty with disabilities also had worse experiences than their non-disabled counterparts when it came to sleep and well-being, both during the remote and in-person periods. As the number of educated individuals who self-identify as having a disability continues to increase in our society [40], and as efforts are being made to ensure a more inclusive work environment (e.g., equity, diversity, and inclusion (EDI)), barriers still appear to exist, and these should be further studied [41,42].

## Figures and Tables

**Table 1:** Age distribution of participants in the study.

Age Range	Faculty	Non-Teaching Staff
55 and over	9	6
45–54	4	8
35–44	8	4
25–34	1	3

**Table 2:** Number of participants’ disabilities.

Disability	Number of Disabilities Reported by Faculty	Number of Disabilities Reported by Non-Teaching Staff	Total Number of Disabilities Reported
Chronic health	4	7	11
Attention deficit hyperactivity disorder (ADHD)	3	2	5
Sensory	4	1	5
Mental health	1	2	3
Autism	0	2	2
Learning disability (Specific learning disorder)	1	1	2
Mobility	1	0	1
Prefer not to say	0	1	1

Note. The 19 participants with a disability indicated 30 disabilities; Of the 19 participants, 14 reported a single disability and 5 reported 2 or more disabilities.

**Table 3:** Sleep and well-being coding categories.

Sleep or Well-Being, Remote and In-Person	Category Includes
Anxiety/Stress	COVID-related concerns associated with the virus
Commute	Commuting/travel to and from college
Work	Teaching, technology, administration, and workplace social interaction
Lifestyle	Family circumstances, work–life separation, routine, leisure time, and social interaction outside work
General	Relevant responses that did not fit into the other categories

**Table 4:** Sleep states-of-mind (SOM) ratios.

	Remote		In-Person	
Group	Faculty	Staff	Faculty	Staff
Disability	0.458	0.491	0.403	0.450
No disability	0.469	0.615	0.477	0.379

**Table 5:** Well-being states-of-mind (SOM) ratios.

	Remote		In-Person	
Group	Faculty	Staff	Faculty	Staff
Disability	0.425	0.565	0.449	0.381
No disability	0.542	0.535	0.421	0.460

## Data Availability

The data are confidential and, therefore, not available to the public.
